# Plasmonic mode interferences and Fano resonances in Metal-Insulator- Metal nanostructured interface

**DOI:** 10.1038/srep14419

**Published:** 2015-09-24

**Authors:** Rana Nicolas, Gaëtan Lévêque, Joseph Marae-Djouda, Guillame Montay, Yazid Madi, Jérôme Plain, Ziad Herro, Michel Kazan, Pierre-Michel Adam, Thomas Maurer

**Affiliations:** 1 Laboratoire de Nanotechnologie et d’Instrumentation Optique, ICD CNRS UMR 6281, Université de Technologie de Troyes, CS 42060, 10004 Troyes, France; 2 Laboratoire de Physique Appliqué (LPA), Université Libanaise, Faculté des Sciences 2, Campus Fanar - BP 90656 - Jdeideh - Liban; 3 Institut d’Electronique, de Microélectronique et de Nanotechnologie (IEMN, CNRS-8520), Cité Scientifique, Avenue Poincaré, 59652 Villeneuve d’Ascq, France; 4 Laboratoire des Systèmes Mécaniques et d’Ingénierie Simultanée, ICD CNRS UMR STMR 6281, Université de Technologie de Troyes, CS 42060, 10004 Troyes, France; 5 Ermess, EPF-Ecole d’Ingénieurs, Sceaux, France; 6 Centre des Matériaux, Mines ParisTech, UMR CNRS 7633, BP 87, 91003 Evry Cedex, France; 7 Department of Physics, American University of Beirut, Riad El-Solh 1107 2020, Beirut, Lebanon

## Abstract

Metal-insulator-metal systems exhibit a rich underlying physics leading to a high degree of tunability of their spectral properties. We performed a systematic study on a metal-insulator-nanostructured metal system with a thin 6 nm dielectric spacer and showed how the nanoparticle sizes and excitation conditions lead to the tunability and coupling/decoupling of localized and delocalized plasmonic modes. We also experimentally evidenced a tunable Fano resonance in a broad spectral window 600 to 800 nm resulting from the interference of gap modes with white light broad band transmitted waves at the interface playing the role of the continuum. By varying the incident illumination angle shifts in the resonances give the possibility to couple or decouple the localized and delocalized modes and to induce a strong change of the asymmetric Fano profile. All these results were confirmed with a crossed comparison between experimental and theoretical measurements, confirming the nature of different modes. The high degree of control and tunability of this plasmonically rich system paves the way for designing and engineering of similar systems with numerous applications. In particular, sensing measurements were performed and a figure of merit of 3.8 was recorded ranking this sensor among the highest sensitive in this wavelength range.

Surface plasmon polariton (SPP) and Localized surface plasmon (LSP) have attracted numerous researchers due to their high technological potential. SPP’s are surface waves confined near a metal dielectric interface that can propagate over large distances^1^, making them appealing for applications in biosensing^2^,^3^. On the other hand LSP resonances can be defined as the localized resonance condition that massively enhances the electromagnetic field in the vicinity of a metal nanoparticle (NP), when the NP have dimensions much smaller than the excitation wavelength^4^. LSP resonance is very sensitive to changes in the NP’s dimensions, the dielectric constant of the surrounding media and the nature of the substrate. Because of intense local electrical field enhancements and sharp resonance excitation peaks, metallic NPs are of great interest for applications in surface enhanced Raman spectroscopy (SERS)^5^, chemical and biological sensors^3^,^6^, cancer treatment^7^ and light harvesting^8^,^9^,^10^. Recently, strong attention was paid to the potentials of SPP and LSP combinations by investigating metallic NPs on top of metallic thin films. Several studies on such systems have indeed shown the coupling and hybridization between localized and delocalized modes, and the effect of the thickness of the dielectric spacer. Those works have revealed that such coupled systems exhibit enhanced optical properties and larger tunability of their spectral properties compared to uncoupled systems^1^,^4^,^11^,^12^,^13^,^14^,^15^,^16^,^17^,^18^,^19^,^20^,^21^,^22^,^23^,^24^. A recent review article on coupled NP/film systems discusses all the advances and the potential applications of these interfaces^25^. Metal NPs/metal film were introduced as super absorbers^26^, optical absorbers using gap plasmon resonators^27^, and controlled reflectance surfaces through gap modes^28^. In addition, several groups have investigated the limit where the spacer layer is subnanometer with trilayer graphene^29^ or even absent^20^, and revealed a plasmonic mode around 520 nm almost independent on the NP’s diameter or the grating periodicity, showing high potential for sensing applications with a figure of merit (FOM) ranging from 2 to 2.9.

Variations in NPs diameters and periodicities as well as the spacer layer thickness and excitation conditions has a direct influence on the wavelength of plasmonic modes, as well as their nature. Our nanostructured metal-insulator-metal (MIM) interface consists of a 6-nm-thick SiO_2_ spacer sandwiched in between a grating of Au NPs and a 50-nm-thick Au film. It is a rich plasmonic playground supporting several plasmonic modes of different nature in a relatively small spectral window. First, it shows two LSP modes: the first is the previously reported 520 nm mode^12^,^28^, characterized by hot spots on top of the NPs, while the longest-wavelength mode is the parallel-dipolar plasmon, strongly shifted to the red by the interaction with the substrate, where the field is enhanced at the bottom of the NPs in contact with the substrate. Second, two SPP modes (one propagating along each side of the 50-nm-thick gold film) can be excited at wavelengths dependent on the grating periodicity and the angle of the incident planewave. Finally, a resonance appears in a wavelength window ranging from 600 to 800 nm, depending on the NPs diameters. As opposed to conventional plasmonic resonances that are described by a lorentzian form, assymetric Fano profiles have been increasingly reported in plasmonic systems with fairly complicated NPs geometries^30^,^31^,^32^,^33^,^34^,^35^. Fano resonances can be described as a general interference process between a continuum or spectrally broad mode acting as a background, and a localized, narrow mode. The key parameter to observe Fano resonance in plasmonic systems is to engineer structures with both a discrete subradiant dark mode and a broad super-radiant one^32^. Several geometries were investigated where the simultaneous excitation of both of these modes was possible. The first investigation of the plasmonic Fano profile was for a dilman-type slab arrangement^36^. Shortly after Fano profile was reported in non-concentric ring disk cavity configuration^37^,^38^. Such interference shows an asymmetric line shape for the total response, where the degree of asymmetry depends on the interference conditions^29^. The Fano lineshape can be represented using the following equation^39^:


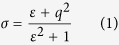


Where σ is the resonance profile of the scattering cross section, q or what is known as the Fano parameter is a phenomenological shape parameter that defines the degree of asymmetry and ε the reduced energy defined by: ε = 2(E–E_F_)/Г where E_F_ is the resonant energy, and Г the width of the auto ionized state. When 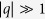 the interference is dominated by the discrete resonance; this results in a Lorentzian peak on top of a weak background. When 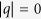 the interference results in a spectral dip and an anti-resonance in the continuum, finally when q = ±1 it indicates that the degree of assymetry is maximum.

The originality of this work is in the simplicity of the designed interface leading to Fano resonances, the 50-nm Au film acts as a mirror with the white light broad band transmitted waves playing the role of the continuum, whose interaction with the localized gap mode in the 6 nm spacer under the gold NPs lead to Fano profiles with a high degree of tunability depending on the NPs diameters and periodicities. Angle-resolved measurements were performed, and the incident angle of illumination was varied from 0° (normal incidence) up to 60°. Spectral shifts of SPP modes and excitation of new modes were reported, resulting in an overlap of SPP with LSP at specific angles. The asymmetry of the Fano profile also decreased with the increase in the angle, where the changes in the excitation condition can change both the amplitude and the phase of the gap mode and the broad continuum thus changing the interference conditions. Finally we showed with sensing measurements that such a MIM interface exhibits a high FOM of 3.8 which ranks this sensor among the highest in this wavelength.

## Results

### Sample preparation

**Figure 1:**
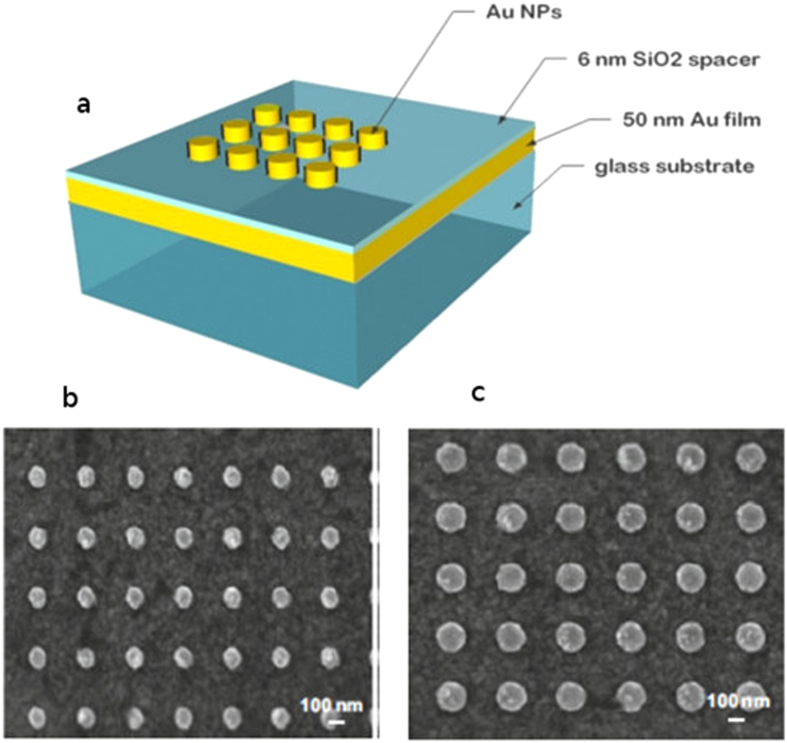
(**a**) Schematic of the designed interface of Au NPs on 6-nm-thick SiO_2_ spacer on a 50-nm-thick Au film. SEM images of Au NPs with center-to-center distance of 300 nm and diameters (**b**) 110 nm (**c**) 140 nm.

The MIM interface was prepared with a grating of Au nano-cylinders having a height of 50 nm, a set of diameters of 80, 110, 140, 170 and 200 nm, and a set of variable periodicities of 300, 450 and 600 nm deposited using electron beam lithography on a 6-nm-thick SiO_2_ film on top of a 50-nm-thick Au film using a 3 nm Cr as an adhesion layer. Figure 1 shows a schematic of the designed interface as well as scanning electron microscopy images confirming the geometry of the NP gratings.

### Optical measurements

**Figure 2:**
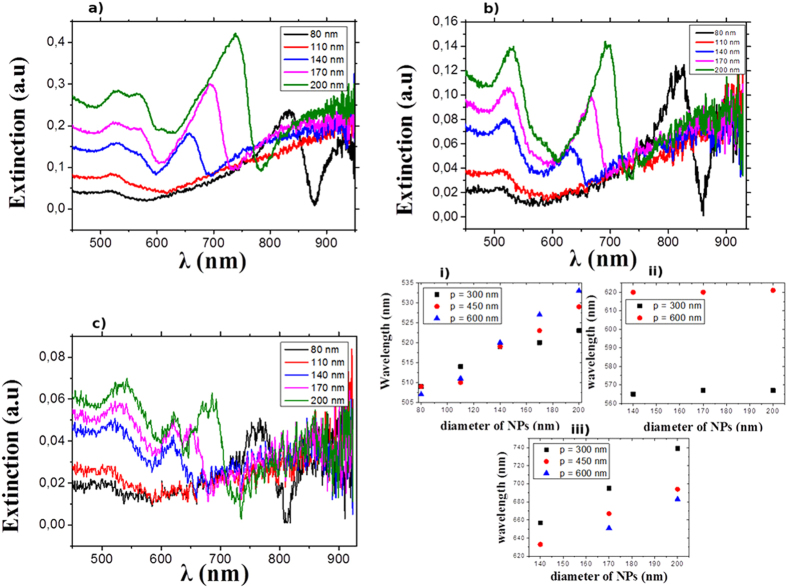
Extinction spectra in transmission and under normal incidence for different diameters of Au NPs (80, 110, 140, 170 and 200 nm) with center-to-center distances of (a) 300 nm (b) 450 nm and (c) 600 nm. Resonance wavelength versus the diameter of NPs for the three different periods (300, 450 and 600 nm): i) for the localized mode at low wavelengths around 520 nm, ii) for the delocalized mode at higher wavelengths between 560 and 620 nm and iii) for the Fano resonance.

Figure 2(a–c) displays the extinction spectra under normal incidence for the different gratings with 300, 450 and 600 nm periodicity. As seen in Fig. 2, these systems are clearly a favorable playground for coupling and hybridization of different plasmonic modes. A very interesting feature of our system is the asymmetric Fano profile resonance between 600 and 800 nm depending on the NP grating dimensions.

The dependence of the different modes on the changes in the diameters of the NPs and the periodicity of the grating is plotted in Fig. 2 (i, ii, iii), which gives an insight on their different nature. The position of the mode around 520 nm, shown in Fig. 2.i, is almost independent on the inter-particle distance but exhibits a slight wavelength linear dependence on the diameter which is an indication^40^,^41^ that it is a localized mode. This is in agreement with previous works done on similar systems by several groups^12^,^16^,^29^ where this mode was identified as localized and independent on the periodicity of the gratings. Fig. 2.ii shows the evolution of the position of a mode whose wavelength is independent on the NPs diameters but dependent on the inter-particle distance; it can be seen around 566 and 620 nm for a periodicity of 300 and 600 nm respectively. However this mode was not observed for the 450 nm periodicity grating. The dependence of the modes on the grating periodicity indicates that this is a delocalized mode excited at different wavelengths for different grating parameters. Figure 2.iii shows the position of an assymetricresonance whose wavelength is linearly dependent on the NPs diameter and blue shifts as the periodicity increases; it has a resonance wavelength which can be tuned between 600 and 800 nm depending on the size and spacing of the NPs. The asymmetry indicates that it is a Fano resonance where fitting the experimental data with Eq. 1 provides a confirmation for the Fano nature of the mode with q values assessed to 1.63 for the NPs of 200 nm diameter and 300 nm periodicity. This corresponds to high asymmetry. Both the delocalized mode and the Fano resonance have not been reported previously but this can be easily explained by the different geometries of the NPs and the different nature and thicknesses of the spacer layer investigated by other groups^12^,^29^,^42^. It is also important to note that Fano resonances observed previously by Lodewijks *et al.* in metal-dielectric-metal interface arise from the coupling of dipole modes^35^ which requires relatively complicated NPs geometries.

However here it arises from the local interference of the gap mode with the broad band continuum of plane waves reflected from the metal film. Fano interferences between LSPR and interface reflections were previously reported for plasmonic systems^43^, but not in a MIM substrate with the localized mode being a gap mode easily tunable by changes in NPs diameters. Finally as expected, a parallel dipolar mode can be seen for the smallest NPs of diameter 80 nm around a wavelength of 820 nm. This mode is red shifted as the NPs diameter increases, which explains why it is not observed for NPs > 80 nm, since their resonance is outside the spectrometer accessible wavelength range.

### Numerical simulations

**Figure 3:**
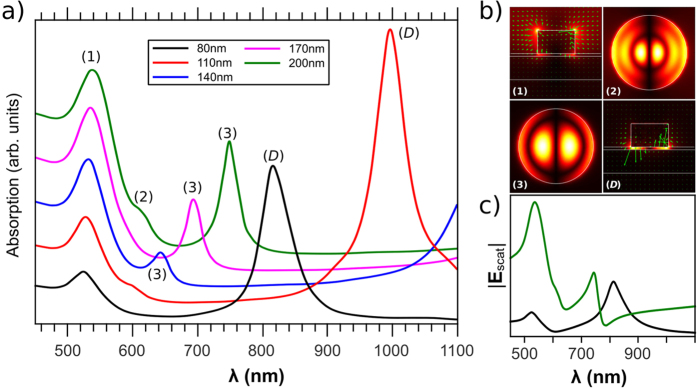
(**a**) Absorption spectra of a single cylinder gold particle of varying diameter on top of the multilayer substrate. The excitation is a TM-polarized plane wave in normal incidence, from the air side. (**b**) Distribution of the electric-field intensity for modes indicated on spectra (**a**): the distribution is plotted in the polarization plane for modes (1) and (*D*), and in a plane parallel to the substrate just under the bottom surface of the cylinder for modes (2) and (3). Maps with the same label on the spectra (**a**) have similar field distributions; the green arrows on maps (1) and (*D*) show the real part of the electric field. (**c**) Amplitude of the field scattered at infinity in the direction of the transmitted incident wave (0°) for particles with diameters 80 nm (black) and 200 nm (green), which slowly vanishes asymptotically.

For a complete analysis of the measured plasmonic modes and the origin of the observed Fano resonance, numerical simulations were performed on both isolated particles and gratings. We have first investigated using the Green’s tensor formalism^44^, the plasmon properties of a single gold cylinder separated by a 6-nm-thick silica spacer from a 50-nm-thick gold film, excited from the air by a TM-polarized plane wave (magnetic field parallel to the surface of the substrate) in normal incidence. The cylinder has a height of 50 nm, a diameter equal to 80, 110, 140, 170 or 200 nm. The absorption spectra of the metal nanoparticles are plotted as a function of the wavelength on Fig. 3(a) for the different diameters, and field distributions for the main resonances are given on Fig. 3(b). The maxima correspond to the excitation of localized surface plasmon modes or plasmonic gap-modes in the coupled particle-film system. First, a common feature between all the spectra is a low wavelength mode around 520 nm, labeled (1), slightly dependent on the NP diameter, in perfect agreement with the experimental results. This mode does not correspond to a vertical LSP, since vertical LSPs cannot be excited in normal incidence due to their symmetry. However, in a previous publication^29^, our group have shown that it is characterized by two hot spots on top of the NP (air side), thus it resembles a dipolar mode localized on the upper surface of the NP, as indicated on the field distribution number (1) on Fig. 3(b). The mode (*D*) is observed for the 80 nm diameter NPs at a wavelength of 815 nm (with a 5 nm discrepancy from the experimental results). The distribution of the electric field of this mode confirms its nature as the commonly observed dipolar plasmon parallel to the film. It is known that this mode is red-shifted when the particle diameter increases, hence for larger diameter its wavelength is beyond 900 nm. Besides these two commonly observed features, our simulations show another highly interesting mode, labeled (3), between 600 and 800 nm depending on the nanoparticle’s diameter. Similarly to mode (*D*), the electric field is essentially concentrated in the portion of the thin silica film under the nanoparticle. The corresponding map in Fig. 3(b) shows the field transverse distribution just under the bottom of the particle, and shows a clear interference pattern. Actually, this gap-mode originates from the fact that the area of the spacer just under the nanoparticle behaves like a resonant cavity for SPP modes. Indeed, the incidence plane wave excites by diffraction a delocalized metal-insulator-metal SPP (MIM-SPP) which can propagate in between the two metal-dielectric interfaces formed by the top of the gold film and the bottom of the gold nanocylinder. Due to the strong impedance mismatch at the NP’s edge, this MIM-SPP bounces back and forth under the cylinder and forms resonant patterns for specific wavelengths. Consistently with that description, the wavelength of that gap-mode increases with the NP’s diameter, from 645 nm for 140 nm diameter to 750 nm for 200 nm diameter.

Furthermore, a higher order gap-mode, labeled (2) on Fig. 3(a) can be seen as a shoulder in the absorption spectrum of the 200 nm particle at 610 nm, whose nature is confirmed by the corresponding field distribution. Finally, the origin of the Fano profile cannot be fully explained by simulations. Fig. 3(c), shows the amplitude of the field scattered by the single NP in the direction of the transmitted incident planewave for 80 nm and 200 nm diameters. The fact that the peak of the dipolar mode at 815 nm for the 80 nm particles is symmetric is an indication that the Fano profile experimentally observed on Fig. 2(a) is the result of the interference of the field scattered by the particle with the transmitted incident planewave. However, a Fano profile similar to the experimental one is shown for the 200 nm particle at the position of the mode number (3). The Fano profile at 740 nm might be attributed to the interference between the corresponding gap mode and the lowest energy dipolar mode, which is at a resonance wavelength beyond our wavelength window. As that mode has an enhanced bandwidth due to its large radiative losses, it should be still intense enough to interfere with the gap mode.

**Figure 4:**
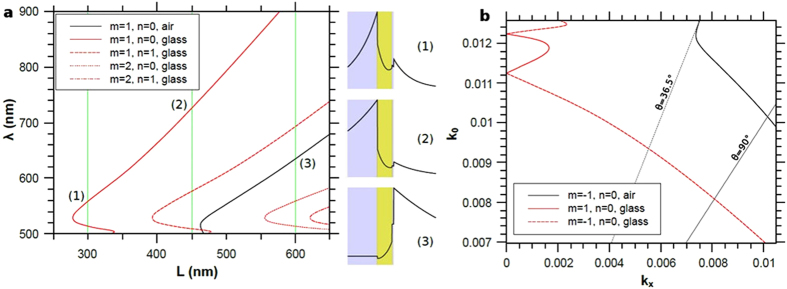
(**a**) Evolution with the period of the excitation wavelength of the two PSP supported by the substrate: black: air-side PSP, red: silica-side PSP. The incident planewave arrives normally to the interface, from the air. On the right are plotted the profile of the electric field for different wavelengths indicated by the numbers. (**b**) Folded dispersion curves of the two PSP modes associated to the substrate, for a period of 300 nm. The wavelength λ in vacuum varies from 500 nm to 900 nm, k_0_ = 2π/λ and k_x_ is the parallel component of the incident wavevector.

Next, we show the properties of the delocalized plasmon modes associated with the substrate, as they can be coupled to the incident light with the grating effect. The substrate alone (without the gold nanocylinders) supports two SPP modes, one for which the light intensity is maximum on the air side (actually just above the thin overcoating glass layer), and the other where the light intensity is maximum on the silica side of the gold film (see the field profile on Fig. 4(a). In the weak coupling regime, the excitation condition is obtained by folding the dispersion curves of the two SPPs in the first Brillouin zone, following:


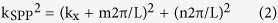


where k_SPP_ is the wavector of the considered SPP modes, m and n are integers and L the periodicity of the grating; the incident planewave is tilted along one of the grating main axis, labeled *x* here, and has a parallel component k_x_ of the wavevector along the interface. In order to compare with the first set of experiments (see Fig. 2), Fig. 4(a) shows the evolution of the SPPs excitation wavelengths in normal incidence (k_x_ = 0), as a function of the period of the grating. We can see that for the periods of 300 and 450 nm, only the SPP on the silica side can be excited, at respectively 560 and 720 nm. The mode at 560 nm for 300 nm periodicity is in perfect agreement with the observed delocalized modes in the experimental results. While for the 450 nm periodicity the resonance wavelength of the SPP at 720 nm spectrally overlaps with the Fano resonance, this explains why it was not observed experimentally. This overlap of two narrow modes, the silica SPP and the gap mode, and their interference with the broad continuum resulted in sharper Fano resonance compared to those measured for different periodicities. This highlights the importance of the nature and intensity of the modes interfering in a Fano profile on the sharpness and degree of asymmetry of the Fano profile. Finally the air-side SPP can be excited for a grating period of 600 nm at λ = 625 nm, close to the experimental wavelength of 635 nm. In order to compare with the next set of experiments, Fig. 4(b) shows the folded SPP curves in the first Brillouin zone for a period of 300 nm, still in the weak coupling regime. For small incidence angles, only the silica-side SPP is excited, the air-side SPP being excited for an incidence angle larger than 36.5° with an incidence wavelength between 500 and 630 nm.

Table 1 shows a comparison of the experimental and numerical results for a grating periodicity of 300 nm and NP diameter of 200 nm, in the case where the excitation light is in normal incidence, see Figs 2(a),3(a) and 4(a). Numerical simulations highly confirm our experimental results for all different diameters and periodicities, and the localized and delocalized modes are all well identified. The interference between the gap mode and a broad white light continuum is observed as Fano profile in the experimental results. It is important to note that up to our knowledge this is the first time a plasmonic Fano resonance with the narrow localized mode being a gap mode is reported; the advantage is the tunability over a wide wavelength range (600 to 800 nm) by a simple variation of the NP’s dimensions.

### Angle resolved measurements

The optical setup we used was modified to tilt the sample, allowing us to investigate illumination and collection angles beyond the traditional normal incidence configuration, up to 60° under TM and TE polarization. Angle-resolved measurements highly enriches this study, since changes in the excitation conditions induce wavelength shift of SPPs allowing a controlled overlap and coupling with localized modes at specific angles. It also allows the observation of new modes that do not appear in normal incidence, an example of which is the air-side SPP that can be excited only at higher angles through folding of the Brillouin zone as previously discussed and shown numerically in Fig. 4(b).

More importantly it enables an ON/OFF switch of the Fano resonance, since the change in the excitation conditions can lead to changes both in the phase and amplitude of the gap mode and continuum, thus inducing changes in the interference profile^43^. This can have numerous interesting applications in sensors, switching, lasing and optoelectronics^32^,^45^,^46^.

**Figure 5:**
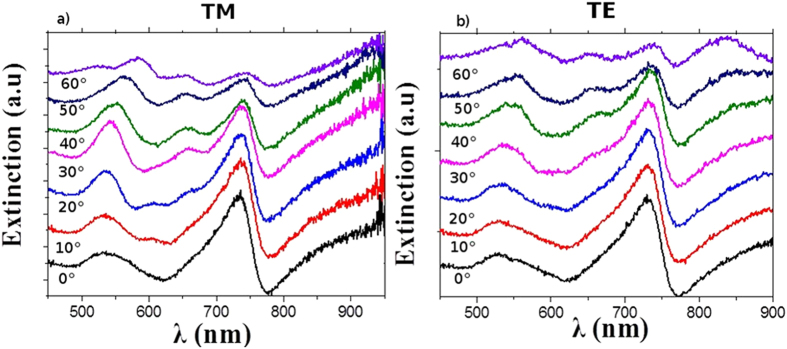
Evolution of extinction spectra versus illumination and collection angles for (a) TE and (b) TM illumination.

Figure 5 shows the evolution of the extinction spectra versus angle for 200-nm diameter NPs with 300-nm periodicity. The extinction was measured as: –log(I_trans_/I_0_), where I_0_ is the transmitted intensity on the substrate on regions without NPs, and I_trans_ the intensity transmitted with on regions with NPs, As expected and shown on Fig. 4(b), the ~560 nm air-side SPP mode red shifts with increasing angle of incidence. These features are more prominent for the TM illumination since SPPs are excited by TM illumination, however the same phenomenon can be seen for TE polarization: here the diffraction process can create the vertical component of the electric field necessary to excite SPPs, even from a TE incident wave. At angles of 30° a perfect overlap between the air SPP and the 520 nm LSP mode is observed with a most intense unique resonance with a full width at half maximum (FWHM) = 17 nm. Both the sharpness and the increase in intensity of this resonance at 30° indicate a coupling and hybridization between the air-side SPP and the LSP. Controlled coupling of localized and propagating modes leading to sharp resonances is very promising for sensing measurements and can increase the sensitivity of such systems by one or two order of magnitudes. Changes in the angles of illumination and collection induce no spectral shifts for gap modes. However the changes in the excitation condition alter both the amplitude and phase of the gap mode and the broad continuum thus changing the interference conditions. It is known that for Fano profiles even minor changes in the strength or phase of the two different interfering modes can change the degree of assymetry^30^. For this reason we observe in our system different lineshapes for the Fano resonance at different angles, were the degree of asymmetry decreases as the angle increases, reaching an almost lorentzian resonance for 60°, with a wavelength matching that of the gap mode on the single NP calculated previously (Fig. 3(a)). The ON/OFF switch of the Fano resonance and the controlled overlap and coupling between LSP and SPP lead to a fundamental understanding and control over the parameters of MIM interfaces; this allows an exact engineering of similar tunable systems favorable for numerous plasmonic and optical applications.

**Figure 6:**
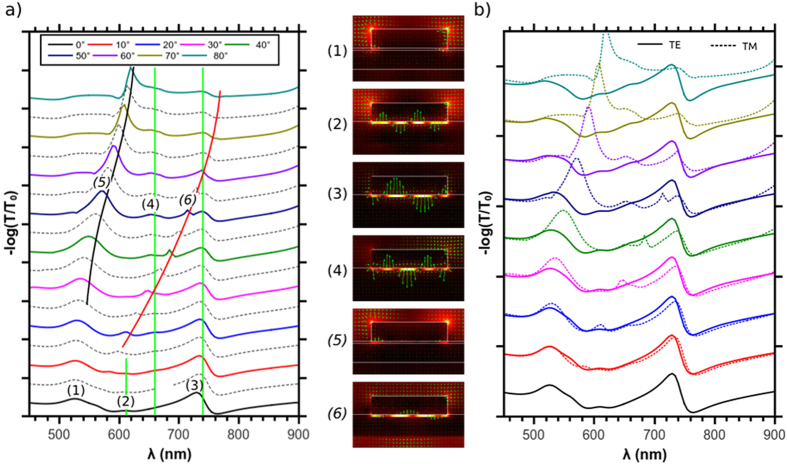
(**a**) Extinction spectra of a 2D square grating of 3D gold nanoparticles of 50 nm thickness and 200 nm diameter on the multilayered substrate, period 300 nm and TM polarization. The solid black and red lines are eye guides for the evolution of the SPP modes on air (black) and glass (red) side. The vertical green lines indicate the position of the non-dispersive gap-modes. The distribution of the electric field amplitude inside the polarization plane has been plotted for few selected angles and wavelengths indicated in the spectra; the green arrows are the real part of the electric field. (**b**) Comparison between the extinction spectra for TM and TE excitation as a function of the incidence angle.

The angle-resolved measurements were supported by numerical simulations. Figure 6(a) shows the angle-dependent extinction spectra computed with a periodic version of the Green’s tensor method^44^,^47^, where the grating has a period of 300 nm, the cylinder NPs have a 200-nm diameter and a 50-nm height. The distribution of the electric field in the polarization plane for selected wavelengths and incidence angles is plotted on the right of Fig. 6(a) (colorscale: electric field amplitude, green arrows: real part of the electric field). The spectra are shown for TM incident polarization; the incident magnetic field is then parallel to the substrate interfaces. A good agreement is obtained with experiments were the localized and delocalized modes as well as the experimental Fano profile are well reproduced. Thus showing the complete picture comparison and matching with experiments, this could not be done with the single NP simulations. In addition new peaks (2) and (4) which appear for large incidence angle in the measurements correspond to the excitation of other gap-modes not seen under normal incidence due to their symmetry; those as well as the gap mode observed for normal incidence (3) are indicated by green lines on Fig. 6(a). The corresponding field distributions are plotted next to the spectra (1) to (6). The air-side SPP(5) is easily seen both in experiments and simulations at incidence angles larger than 40°, however the silica-side SPP (6) is too narrow to be resolved in the experiments. Overall, **c**ompared to the simulated peaks, the experimental ones are a bit wider, which explains that the number of resonances does not always correspond for every incidence angle. This happens particularly when two peaks in the simulations are very close to each other, like for example with the 50° incidence. For that value, two modes are evidenced in the simulations near 720 nm, which are not resolved in the experiment. One corresponds to the grating-excited silica-side SPP (710 nm), while the other corresponds to the Fano profile occurring at the gap-mode number (3) (730 nm). Another important conclusion which results from the comparison between the numerical simulations and experimental measurements is that at higher angles peaks which seem to correspond to the same mode because they have the same wavelength for different angles might differ in nature. Indeed, as shown on Fig. 6(a), the positions of the delocalized modes is extremely angle sensitive, and their position is often very close to the one of the localized modes. For example, the experimental spectra for 20° and 30° incidence angles show both one mode at 655 nm, however they are of different nature, where it corresponds to the localized mode number (4) at 20° and to the substrate-side SPP for 30°, which appears more intensely in the simulations while being close to the localized mode wavelength at this angle. Finally, we can see on Fig. 6(b) that the spectra for the TE polarization are less angle-sensitive in the simulations than in the experiment. A major difference is the intense mode which shows at 850 nm in the experiments for incidence angles larger than 40°. The fact that it is angle dependent is an indication that it might correspond to a delocalized mode, but it could not be reproduced in the simulations.

### Sensing measurements

**Figure 7:**
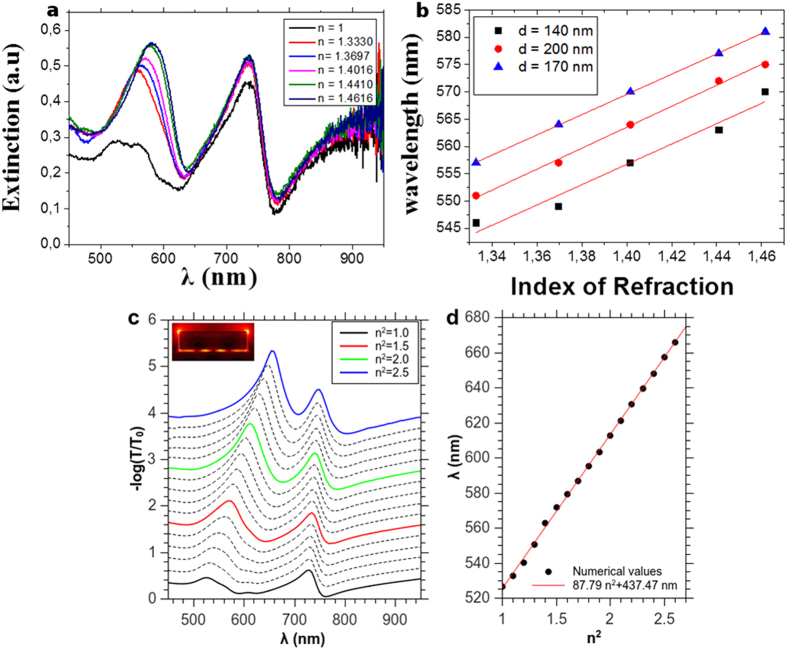
(**a**) Extinction spectra of the Au NPs with 200 nm in diameter and 300 nm in periodicity, for different refractive indexes n of glycerol/water mixtures: 1.00 (black), 1.330 (red), 1.3697 (light blue), 1.4016 (magenta), 1.4410 (green), and 1.4616 (dark blue) (**b**) Shift of the low wavelength LSPR peak as a function of the refractive index of the surrounding medium; (**c**) Numerical simulation of the extinction spectra of the Au NPs with 200 nm in diameter and 300 nm in periodicity, for different dielectric constants n^2^ of the NPs’ surrounding medium; the inset shows the distribution of the amplitude of the electric field in the polarization plane for n = 1.4 and λ = 610 nm; (**d**) Numerical shift of the low wavelength localized plasmon with n^2^ and corresponding linear regression. The refractive index sensitivity for n = 1.4 is equal to 246 nm/RIU.

Finally, the sharp low-wavelength mode encouraged us to perform refractive-index sensing measurements. Bulk index of refraction surrounding the NPs was changed by the use of mixtures of water and glycerol at different concentrations, giving indices of refraction varying from n = 1.33 to n = 1.47. All measurements were performed under normal illumination. Figure 7 presents the evolution of the extinction spectra versus the increase in the index of refraction of the surrounding medium. This experiment shows how the increase in the index of refraction induces a red shift of the LSP mode and a blue shift of the glass-side SPP mode, leading to a coupling of these modes and a sharp resonance around 550 nm, linearly dependent on the change in index of refraction. However the gap mode is almost insensitive to changes in the index of refraction which could be expected since the energy is stored in the gap. Sensitivity of LSP sensors is defined as the shift in the wavelength versus the change in the index of refraction and it was calculated for NPs with different diameters and a 300 nm grating periodicity (Fig. 7(b)). Sensitivities of 186, 192 and 185 nm/RIU (nanometers per refractive index unit) were recorded for the 140, 170 and 200 nm NPs respectively.

These sensitivities are remarkably higher compared to similar NPs/film systems without a spacer layer. On the other hand, this peak profile is significantly sharpened compared to the case were no metal film is present and its full width at half maximum (FWHM) is equal to 50 nm. The figure of merit (FoM) is defined as the ratio of sensitivity to FWHM and is commonly used to quantify the resolution of LSP sensors. Thus the two advantages of the metal-insulator–metal systems in increasing the sensitivity and in sharpening the mode spectral width are very promising for enhancing the plasmonic sensors. The FoM for the three different diameters of NPs was calculated. The 170 nm NPs recorded the highest FoM of 3.76. Such a FoM value is higher than those reported for silver triangles (λ_peak_ = 564 nm; FoM = 1.8)^48^, silver cubes (λ_peak_ = 510 nm; FoM = 1.6)^49^, silver spheres (λ_peak_ = 520 nm; FoM = 2.2)^50^ or gold spheres λ_peak_ = 530 nm; FoM = 1.5)^51^. It is also higher than FoM reported for the same 520 nm mode when the spacer layer is trilayer graphene FOM (2.9) or absent(2.1)^29^, thus achieving a 29%, and a 79% increase respectively with respect to similar modes on different systems. This ranks our sensor among the highly sensitive, if not one of the highest LSP sensors with plasmon band in the visible at 500 nm^52^. Indeed, most of the interfaces with high FoM (4–16.5)^53^ take advantage of the fact that higher sensitivities are achieved with plasmon bands in the near-infrared of the spectrum (850–1,200 nm). Finally, numerical simulations have been performed for the particle with 200 nm diameter, showing a faster red-shift of the low-wavelength localized plasmon with the index of refraction than in the experiment. Figure 7(c) shows clearly, together with the electric field distribution for n = 1.4 at the low wavelength resonance, that this is the same mode with the two hot-spots at the top edges of the nanocylinder which shifts to the red, while the gap modes are more weakly affected by the change in refractive index, as the light is concentrated inside the gap. Figure 7(d) shows the evolution of the position of the low-wavelength mode wich is remarkably linear with n^2^. The refractive index sensitivity is 246 nm/RIU for that mode around n = 1.4, while the gap mode at 740 nm has a lower sensitivity of about 38 nm/RIU. The discrepancy between the numerical and the experimental value can be explained by the fact that the edges are sharper in the model than in the experiment, where the shape of the particle is probably slightly conical.

## Conclusion

In this work we have investigated a metal-insulator metal (MIM) system, where localized and delocalized plasmonic modes as well as Fano resonances were measured in a relatively small spectral window 500 to 850 nm. A systematic study of the effect of the size of the NP and the periodicity of the grating in tuning these resonances was performed. Simulations were highly in agreement with the experimental results, and electric field maps confirmed the nature of the different observed modes. Inducing Fano resonances that are easily tunable in this relatively simple plasmonic substrate is remarkable especially that most previous work on plasmonic Fano resonances investigate the coupling of LSPR modes with dark dipolar modes which require complicated NPs geometries. Angle resolved measurements were also performed both experimentally and numerically, were the high dependence of SPP resonances on excitation conditions allowed a controlled overlap between air-side SPP mode and the 520 nm LSP mode with an intense sharp mode highly interesting for sensing measurements. Also the change in the excitation condition altered the asymmetry of the Fano profile as the interference conditions changed with the increased angle and an ON/OFF switch of the Fano resonance was achieved. Finally sensing measurements were performed as a direct application and a high FOM of 3.8 was recorded placing our sensor among the highest in this wavelength range with a 79% increase compared to sensors based on the same dipolar mode.

## Methods

Using electron beam deposition, a 50 nm Au film was evaporated on a glass substrate followed by the evaporation of a 6 nm SiO_2_ layer, a 3 nm Cr adhesion layer was used to avoid common adhesion problems between Au and SiO_2_. A 150 nm layer of poly (methylmethacrylate) (PMMA) was later deposited by spin coating (at 4000 rpm) and then annealed during 15 min at 150 °C. Finally Electron Beam Lithography (EBL) was used to prepare 15 zones of gold NPs arrays with well-defined structure. Depositing the NPs on a metallic film was advantageous since the charging effect common for EBL on dielectric substrates was avoided by charge dissipation in the Au film. The extinction spectra of the systems have been measured with a transmission optical microscope coupled to a micro-spectrometer using a multimode optical fiber as confocal filtering. A × 50 objective lens (NA = 0.30) allows for a detection area of ≈50 μm^2^. The setup has been upgraded to allow angle-resolved measurements.

## Additional Information

**How to cite this article**: Nicolas, R. *et al.* Plasmonic mode interferences and Fano resonances in Metal-Insulator- Metal nanostructured interface. *Sci. Rep.* **5**, 14419; doi: 10.1038/srep14419 (2015).

## Figures and Tables

**Table 1:** Comparison of the numerical and experimental results of all the observed plasmonic modes for nanoparticles having a diameter of 200 nm and a 300 nm periodicity, for excitation in normal incidence.

	Dipolar mode localized on the upper surface of the nanoparticle.	Gap modes (observed as Fano profile experimentally because of the coupling with SPP	Air-side SPP (m = 1, n = 0)
Experimental	~520 nm	735 nm	559 nm
Numerical	~520 nm	738 nm	560 nm
